# Higher serum selenium concentration is associated with lower risk of all-cause and cardiovascular mortality among individuals with chronic kidney disease: A population-based cohort study of NHANES

**DOI:** 10.3389/fnut.2023.1127188

**Published:** 2023-03-31

**Authors:** Daiwen Zhu, Qiang Zhong, Tao Lin, Turun Song

**Affiliations:** ^1^Department of Urology, Institute of Urology, West China Hospital, Sichuan University, Chengdu, Sichuan, China; ^2^Organ Transplantation Center, West China Hospital, Sichuan University, Chengdu, Sichuan, China; ^3^Organ Transplantation Center, Affiliated Hospital of Zunyi Medical College, Zunyi, Guizhou, China

**Keywords:** selenium, chronic kidney disease, antioxidant, oxidative stress, mortality

## Abstract

**Background:**

Selenium is an essential nutrient and trace element required for human health and plays an important role in antioxidative and anti-inflammatory processes. However, the long-term impact of selenium levels on the health of patients with chronic kidney disease remains unclear.

**Method:**

Participants in this study were 3,063 CKD adults from the Third National Health and Nutrition Examination Survey (NHANES 1999–2000, 2003–2004, and 2011–2018). The mortality status and the cause of death of the study participants were obtained from the National Death Index records. For all-cause and cardiovascular disease (CVD) mortality, the models employed to estimate hazard ratios (HRs) and 95% CI were Cox proportional hazard models and competing risk models, respectively.

**Result:**

During the follow-up period, 884 deaths occurred, including 336 heart-disease-associated deaths. The median (IQR) concentration of serum selenium was 181.7 (156.1, 201.5) μg/L. After full adjustment, serum selenium levels were associated with a decreased risk of mortality in patients with CKD, including all-cause and CVD mortality (P < 0.001). The multivariate-adjusted HRs (95%CI) were 0.684 (0.549–0.852) for all-cause mortality (*P*_trend_ < 0.001) and 0.513 (0.356–0.739) for CVD mortality (*P*_trend_ < 0.001) when selenium concentrations were compared according to the extreme quartiles. Selenium levels are inversely associated with an increased risk of all-cause mortality and CVD mortality. Similar results were observed in subgroup and sensitivity analyses.

**Conclusion:**

Higher serum selenium concentration was independently associated with a decreased risk of all-cause and CVD mortality in patients with CKD.

## Introduction

Chronic kidney disease (CKD) is a serious health problem that poses a major threat to over 15–20% of the global general population and has become a significant challenge for society and healthcare systems worldwide (1). Among Medicare patients in the United States, the incidence of CKD has reached 14.5% and is higher in older adults (2–4). Patients with CKD suffer from obviously high morbidity of comorbid cardiovascular diseases (CVDs), including arrhythmias and coronary artery disease (CAD) (1, 5). In the progression of CKD and comorbid CVDs, pro-inflammatory processes, oxidative stress, and vascular endothelial dysfunction can amplify and induce each other (6–8). Among the vicious circuits formed by pathological changes, oxidative stress can promote the development of chronic inflammation in patients with CKD and worsen renal injury (6, 9–13).

Selenium, an essential nutrient and trace element, plays a crucial role in anti-inflammatory and antioxidant processes (14). In the general population, meat and eggs are important dietary sources of selenium, whereas flour and rice are alternative dietary sources (15). Dietary selenium is absorbed in the intestinal tract and transformed into different metabolites through various metabolic pathways (16–18). Available dietary selenium is determined by the form of selenium (including organic and inorganic forms) and type of food (including meats, grains, and seafood) (16, 19). Insufficient dietary intake of selenium and selenium deficiency are important health challenges that induce Keshan disease, Kashin–Beck disease, autoimmune diseases, and CVDs (14, 20, 21). However, existing evidence reveals that high selenium levels have a beneficial impact on the incidence of various diseases in the general population, whereas supplementation with selenium has demonstrated controversial results (14, 22–33). Among patients with CKD with extremely high pro-inflammatory status and oxidative stress, who always have abnormal metabolisms of various trace elements, investigations regarding the beneficial effects of selenium are controversial and limited. One prospective study suggested that the serum selenium levels of patients with hemodialysis were similar to those of the general population (34), whereas three observational studies revealed that the serum selenium levels of patients with CKD were lower than those of healthy adults (28, 30, 32). Some intervention studies found that selenium supplementation may play a beneficial role in patients with CKD (35, 36), while a randomized controlled trial (RCT) indicated that oral administration of selenium does not decrease the prevalence of type 2 diabetes and leads to an increased risk of this disease during the follow-up period (37). To the best of our knowledge, no study has examined the long-term impact of serum selenium levels on all-cause mortality and CVD mortality in patients with CKD.

In the present study, we prospectively investigated the association of serum selenium levels with all-cause and CVD mortality among patients with CKD from the National Health and Nutrition Examination Survey (NHANES).

## Methods

### Study population

National Health and Nutrition Examination Survey (NHANES) is a nationally representative survey of the US civilian non-institutionalized population that is conducted by the National Center for Health Statistics (38). Since 1999, the NHANES has collected data continuously and released datasets every 2 years. The datasets contain information from personal interviews, physical examination results, and laboratory data. The NHANES cohort has been widely used to explore the associations between nutrients and mortality in the general population and different disease states (38–40). We conducted this cohort study using individuals from the NHANES 1999–2000, 2003–2004, and 2011–2018 cohorts with selenium measures and assessments of CKD.

### Measurement of selenium levels and the diagnosis of chronic kidney diseases

As reported, the measurement method for serum selenium levels was inductively coupled plasma–dynamic reaction cell–mass spectrometry. In detail, the blood samples of participants were collected in containers, and then, the samples clotted and were centrifugated at 1,115 × *g* for 15 min. The serum samples were then stored under the proper freezing conditions and were prepared for transport to the laboratory. Based on the Kidney Disease Improving Global Outcomes (KDIGO) guideline, the estimated glomerular filtration rate (eGFR) and urinary albumin-to-creatinine ratio were used to define CKD (41). Using the Chronic Kidney Disease Epidemiology Collaboration (CKD-EPI) equation, the eGFR of every participant was calculated. CKD was graded as follows: participants with eGFR ≥ 90 ml/min/1.73 m^2^ and albuminuria were categorized into stage 1; participants with eGFR of 60–89 ml/min/1.73 m^2^ and albuminuria were categorized into stage 2; participants with eGFR of 30–59 ml/min/1.73 m^2^ were categorized into stage 3; participants with eGFR of 15–29 ml/min/1.73 m^2^ were categorized into stage 4; and the participants with eGFR of <15 ml/min/1.73 m^2^ were categorized into stage 5.

### Mortality outcome of the study population

The source of mortality information was data collected from the National Death Index, until 31 December 2018. The follow-up time for each participant was examined from the time of participation to the date of death or 31 December 2018. Using the International Statistical Classification of Diseases and Related Health Problems, 10th revision (ICD-10), the underlying cause of death was identified in this database. In the present study, we examined the effect of serum selenium levels on all-cause and CVD mortality. Specifically, codes I00–I09, I11, I13, and I20–I51 were defined as CVD deaths in ICD-10.

### Covariates assessment

All data were obtained from the NHANES 1999–2000, 2003–2004, and 2011–2018 cohort databases. Demographic characteristics included in the present study were age, sex, race, family poverty-to-income ratio, body mass index (BMI), and smoking status. Serum triglyceride, total cholesterol, and uric acid concentrations were obtained from laboratory test results. Data on diabetes and hypertension were obtained as chronic comorbidities. Using total family income divided by the poverty threshold, the family poverty-to-income ratio was calculated. The smoking status of every participant was measured. The study population was categorized into smokers (smoking now or not smoking now but >100 cigarettes in life) and never smokers (<100 cigarettes in life). Diabetes was diagnosed according to a self-reported doctor's diagnosis of diabetes or laboratory test results. Positive laboratory test results included glycated hemoglobin (HbA1c) of ≥6.5%, fasting blood glucose of ≥7.1 mmol/L, random serum glucose of ≥11.1 mmol/L, and 2-h serum glucose of ≥11.1 mmol/L based on oral glucose tolerance tests.

### Statistical analyses

Serum selenium concentrations were classified into four categories according to quartiles. Continuous variables are expressed as the mean (standard deviation), while categorical variables are expressed as numbers (proportions). Continuous and categorical demographic variables were compared using the analysis of variance (ANOVA) and the chi-square test.

First, we examined the correlation between serum selenium levels and mortality in patients with CKD using restricted cubic spline analyses. The Kaplan–Meier model analyses were conducted to estimate the cumulative incidence of all-cause death, whereas competing risk model analyses were employed to estimate the cumulative incidence of cardiovascular death. We examined the impact of different serum selenium levels on all-cause mortality using the Cox proportional hazards analysis models, whereas we investigated the impact of different serum selenium levels on cardiovascular-cause mortality using competing risk models. In model 1, the estimated models were adjusted for variables including age, sex, and race. In model 2, based on model 1, the estimated models were further adjusted for family income–poverty ratio, BMI, serum triglycerides, serum total cholesterol, and serum uric acid. In model 3, based on model 2, the estimated models were the fully adjusted models, further adjusted for diabetes, hypertension, and smoking status. In model 4, serum selenium concentrations were analyzed as the continuous variable, and the results were fully adjusted for model 3. To confirm the previous correlation, age (≥65 or <65 years), sex (male or female), BMI (≥30 or <30 kg/m^2^), race (non-Hispanic white or other), smoking status (smoker or never smoker), and various subgroup analyses were performed. Different sensitivity analyses were performed by excluding different subgroups of participants as follows: (1) participants who died within 1 year; (2) participants with CKD stages 3–5, and (3) participants with CKD stages 1–2. All statistical analyses were performed using R version 4.1.0.

## Results

**Figure 1:**
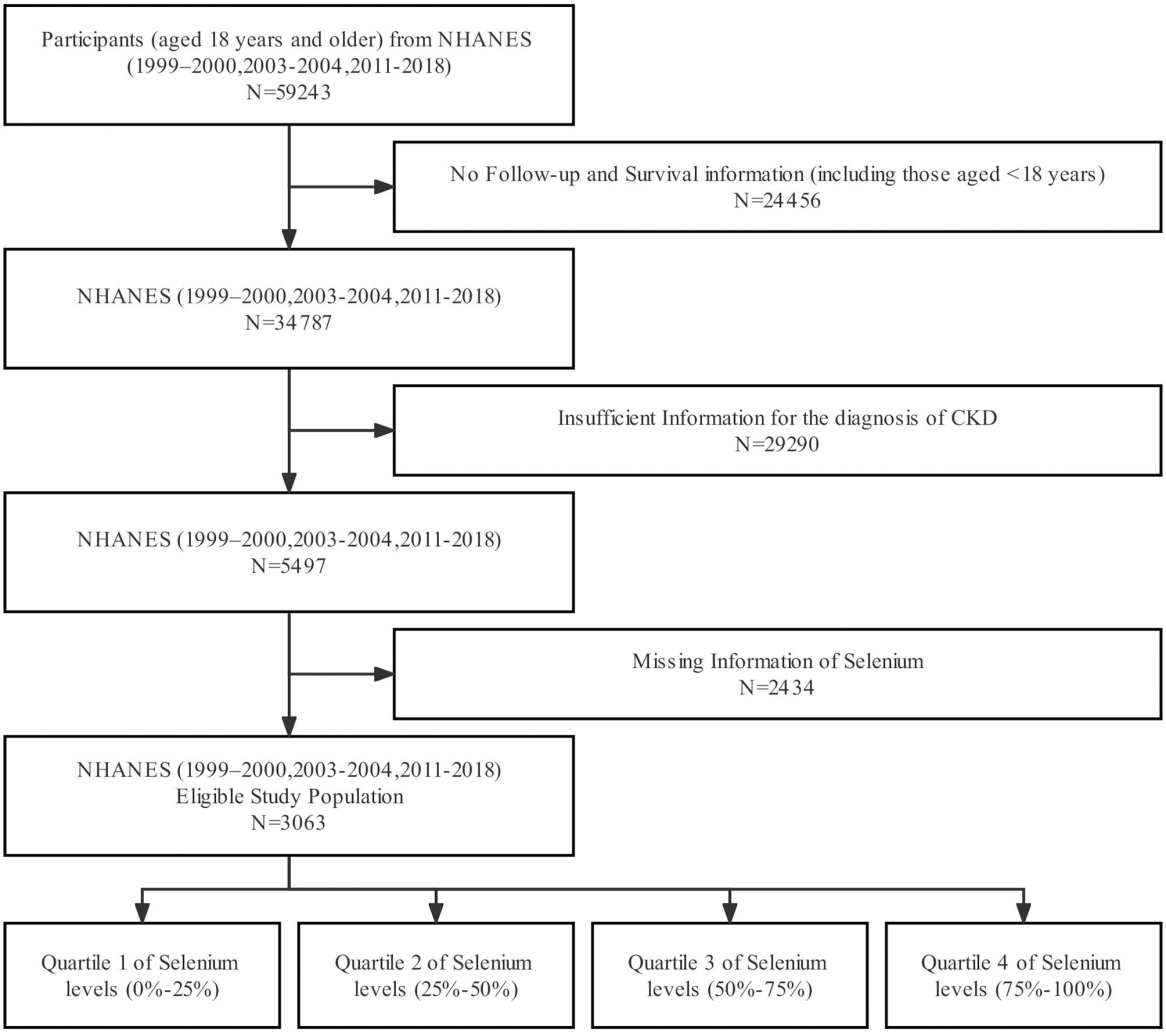
Flow chart.

The current analysis included 3,063 individuals, and the complete preparation process is shown in Figure 1. The baseline characteristics of the study participants based on quartiles of serum selenium concentrations are presented in Table 1. In these participants, the median (interquartile range) serum selenium concentration was 181.7 (156.1–201.5) μg/L; the mean age was 64.0 ± 16.3 years, 48% were male, and 48% were non-Hispanic white. Higher serum selenium levels were associated with participants who were male, younger, of other races (different from non-Hispanic white), never smokers, and with a higher eGFR.

**Table 1:** Baseline characteristics of participants with CKD according to serum selenium in NHANES (1999–2000, 2003–2004, and 2011–2018)^a^.

	**Serum selenium concentration (**μ**g/L)**					
**Characteristics**	**Total**	**Q1**<**156.1**	**Q2** **156.1–181.7**	**Q3** **181.7–201.5**	**Q4** **201.5–734.8**	***P***
Number of patients, *n*	3,063	766	768	762	767	
Sex (%)						
Male	1,478 (48)	354 (46)	357 (46)	352 (46)	415 (54)	0.003
Female	1,585 (52)	412 (54)	411 (54)	410 (54)	352 (46)	
Age, years	64.0 (16.3)	70.0 (13.0)	63.5 (17.1)	61.5 (17.2)	60.9 (16.2)	<0.001
Ethnicity (%)						
Non-Hispanic white	1,455 (48)	449 (59)	331 (43)	327 (43)	348 (45)	<0.001
Other	1,608 (52)	317 (41)	437 (57)	435 (57)	419 (55)	
Family income–poverty ratio	2.3 (1.5)	2.3 (1.5)	2.2 (1.5)	2.2 (1.5)	2.4 (1.6)	0.148
Family income–poverty ratio (%)						
>3.0	896 (29)	222 (29)	226 (29)	209 (27)	239 (31)	0.11
1.1–3.0	1,478 (48)	396 (52)	354 (46)	373 (49)	355 (46)	
≤1.0	689 (22)	148 (19)	188 (24)	180 (24)	173 (23)	
BMI, kg/m^2^	29.9 (7.1)	28.9 (6.5)	29.9 (7.5)	30.5 (7.3)	30.3 (7.2)	<0.001
Smoke status (%)						
Never smoker	1,516 (49)	348 (45)	384 (50)	403 (53)	381 (50)	0.034
Smoker	1,547 (51)	418 (55)	384 (50)	359 (47)	386 (50)	
Triglycerides, mg/dl	167.8 (128.3)	155.2 (123.8)	151.0 (101.7)	167.0 (116.9)	197.8 (158.9)	<0.001
Cholesterol, mg/dl	192.5 (46.8)	194.7 (46.6)	187.4 (44.0)	188.6 (45.0)	199.5 (50.4)	<0.001
Uric acid, mg/dl	6.0 (1.6)	6.1 (1.6)	6.0 (1.7)	5.9 (1.6)	6.1 (1.6)	0.111
Serum creatinine, mg/dl	1.2 (0.7)	1.2 (0.8)	1.2 (0.9)	1.1 (0.6)	1.1 (0.5)	<0.001
eGFR, mL/min/1.73m^2^	70.2 (28.8)	62.2 (24.9)	69.0 (29.8)	72.6 (29.6)	76.9 (28.8)	<0.001
Urinary creatinine, mg/dl	117.1 (78.0)	112.2 (69.5)	119.6 (82.9)	115.0 (78.2)	121.5 (80.3)	0.078
Urinary albumin, μg/ml	201.2 (664.8)	202.8 (767.0)	226.0 (828.2)	202.2 (559.7)	173.8 (424.0)	0.498
Hypertension (%)						
No	836 (27)	178 (23)	226 (29)	224 (29)	208 (27)	0.02
Yes	2,227 (73)	588 (77)	542 (71)	538 (71)	559 (73)	
Diabetes (%)						
No	1,840 (60)	501 (65)	459 (60)	449 (59)	431 (56)	0.002
Yes	1,223 (40)	265 (35)	309 (40)	313 (41)	336 (44)	
CKD stage (%)						
Stage 1–2	1,543 (50)	304 (40)	374 (49)	410 (54)	455 (59)	<0.001
Stage 3–5	1,520 (50)	462 (60)	394 (51)	352 (46)	312 (41)	

a Continuous variables are presented as means (SD). Categorical variables are presented as numbers (percentages).

Q, quartile; BMI, body mass index; eGFR, estimated glomerular filtration rate; CKD, chronic kidney diseases.

No missing values.

**Figure 2:**
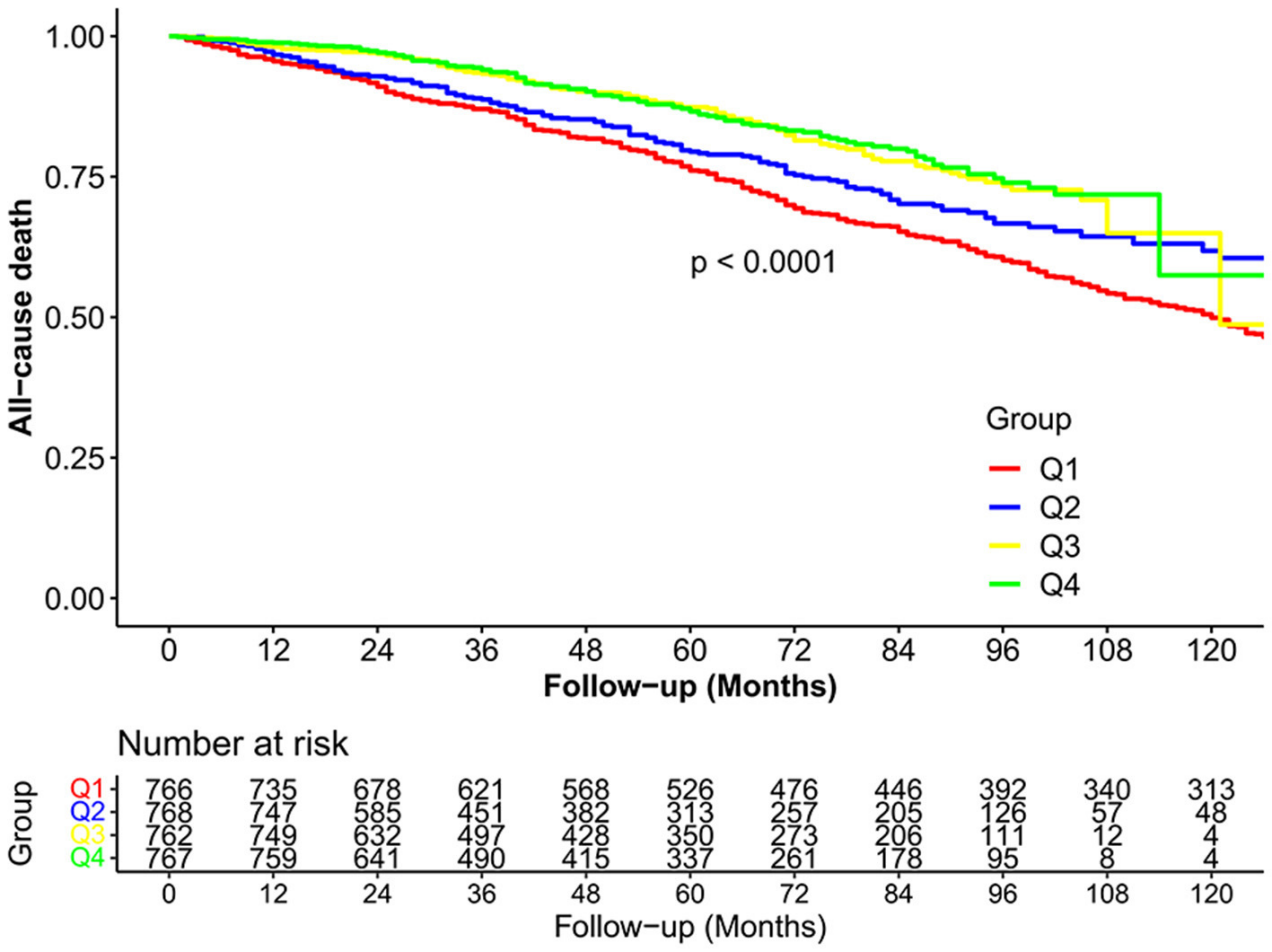
The cumulative incidence of all-cause death in the four study groups during the follow-up period.

**Figure 3:**
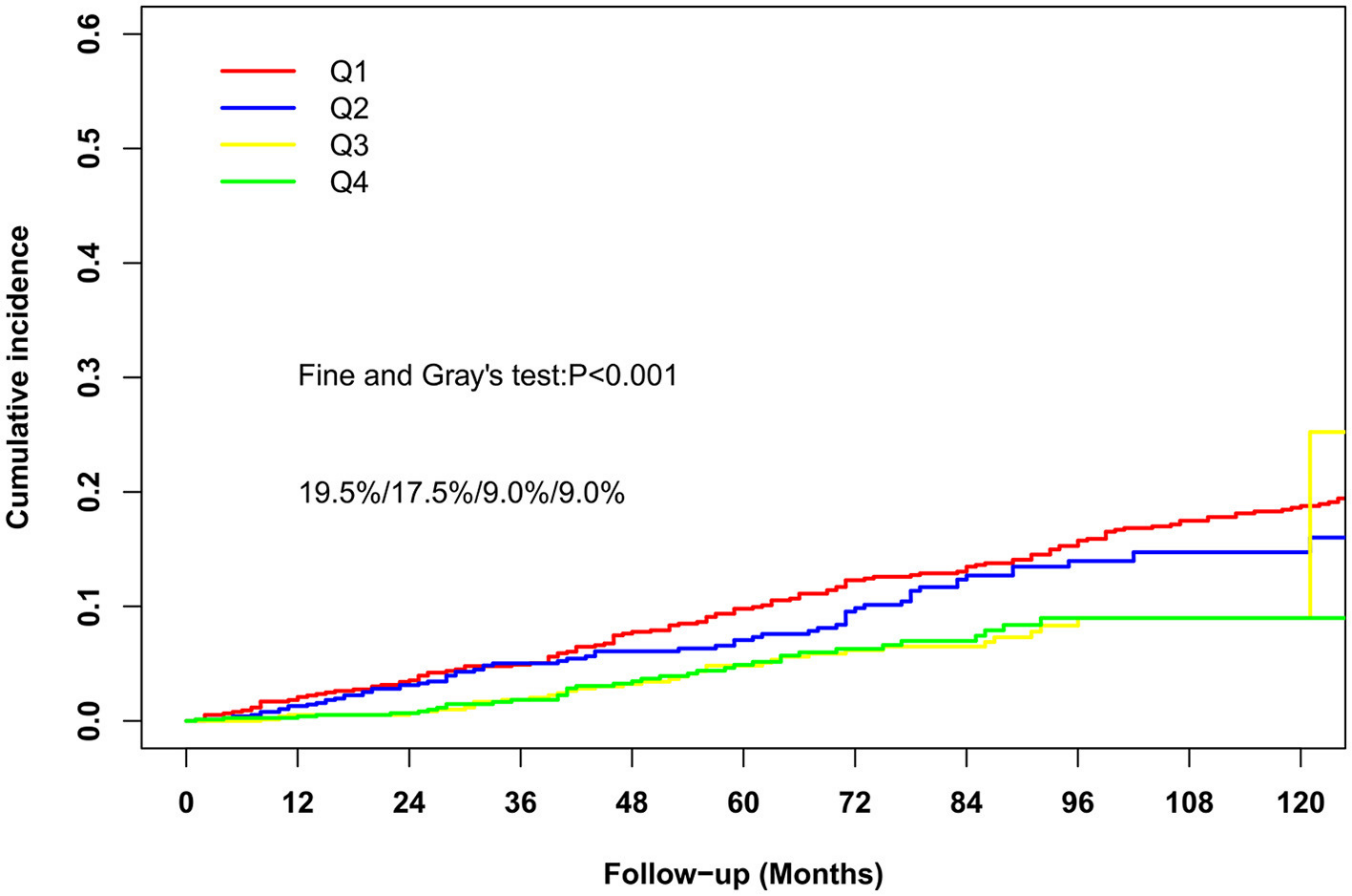
The cumulative incidence of cardiovascular-cause death in the four study groups during the follow-up period.

**Figure 4:**
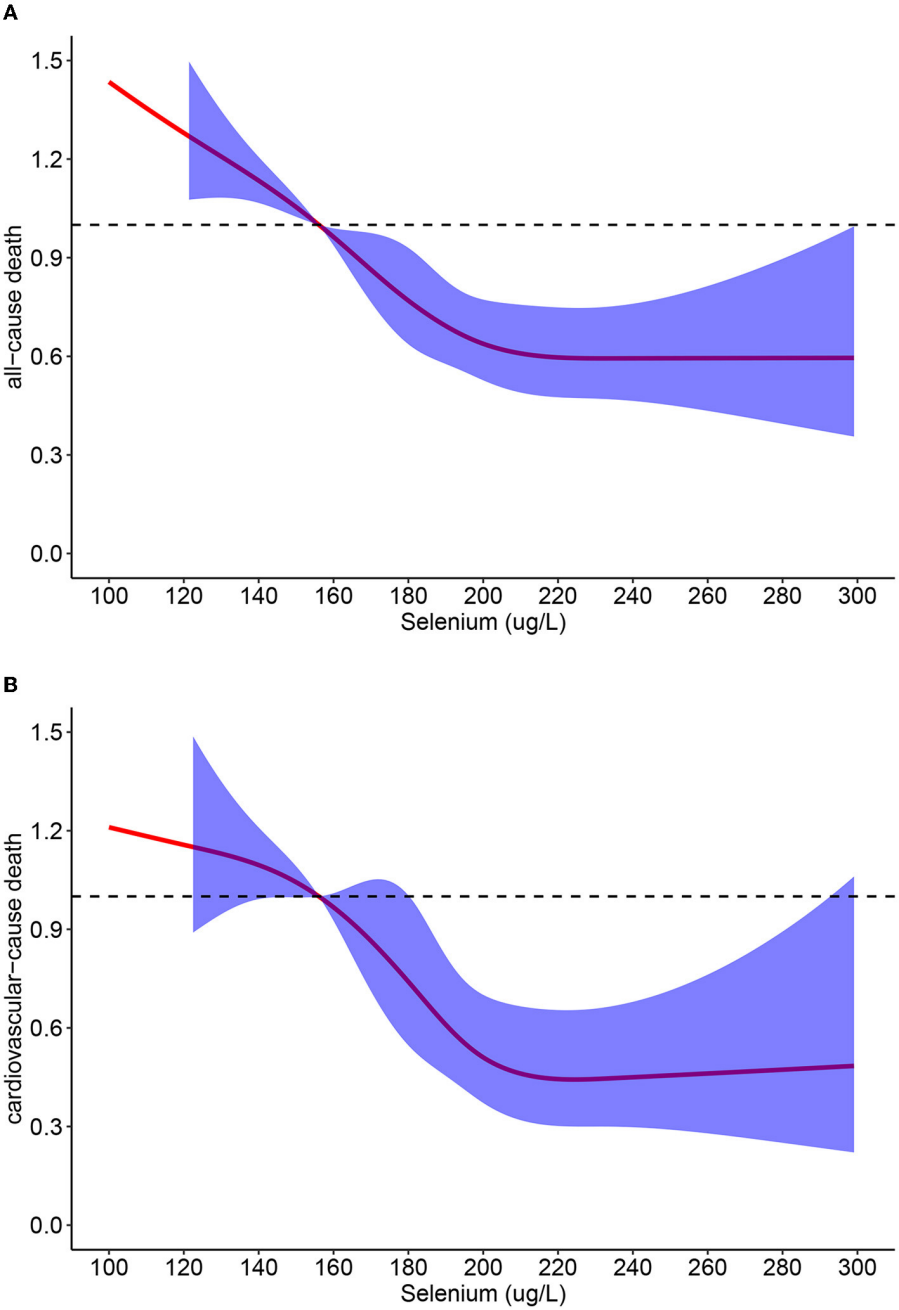
**(A)** Restricted cubic spline analyses between serum selenium concentrations and all-cause mortality; **(B)** Restricted cubic spline analyses between serum selenium concentrations and cardiovascular-cause mortality.

Of the 3,063 participants, during the follow-up period, 884 deaths occurred, including 336 cardiovascular deaths. The median follow-up period was 59 months. The Kaplan–Meier curve revealed that participants with higher serum selenium concentrations had significantly higher survival rates during the follow-up period (*P* < 0.0001; Figure 2). Similar results were observed between selenium concentrations and CVD mortality (Figure 3). The cumulative incidence of cardiovascular death in the different quartiles from Q1 to Q4 was 19.5, 17.5, 9.0, and 9.0%, respectively (*P* < 0.001), with a follow-up time of 120 months. Higher selenium levels were consistently correlated with a reduction in all-cause mortality among participants with CKD after multivariate adjustment (Table 2). As shown in Table 2, after multivariate adjustment, the hazard ratios (HRs; 95% CI) of the different quartiles from Q1 to Q4 were 1 (reference), 0.866 (0.724–1.035), 0.737 (0.596–0.911), and 0.684 (0.549–0.852), respectively (*P*_trend_ < 0.001). In model 4, all-cause mortality decreased by 0.5% for every 1% increase in serum selenium concentration (HR 0.995, 95% CI, 0.993–0.997; *P* < 0.001). A consistent correlation between selenium levels and all-cause mortality was observed according to the restricted cubic spline analysis results (*P* < 0.001; Figure 4A). A similar inverse correlation between serum selenium concentration and CVD mortality was observed, and higher serum selenium levels were correlated with a reduction in CVD mortality (Table 3). After multivariable adjustment, the HRs (95% CI) of the different quartiles from Q1 to Q4 were 1 (reference), 0.834 (0.633–1.100), 0.526 (0.370–0.748), and 0.513 (0.356–0.739; *P*_trend_ < 0.001), respectively. For every 1% increase in serum selenium concentration, the risk of CVD mortality decreased by 0.8% (HR 0.992, 95% CI, 0.989–0.996; *P* < 0.001). A non-linear dose–response relationship between serum selenium levels and CVD mortality was also determined (*P* = 0.001; Figure 4B).

**Table 2:** All-cause mortality according to quartiles of serum selenium concentrations among patients with CKD^a^.

**Characteristics**	**Serum selenium concentration (**μ**g/L)**				
	**Q1**<**156.1**	**Q2 156.1–181.7**	**Q3 181.7–201.5**	**Q4 201.5–734.8**	***P*** **-trend**
All-cause mortality					
Number of deaths/total	478/776	177/768	119/762	110/767	884/3,063
Model 1^b^	1	0.903 (0.756, 1.078)	0.748 (0.605, 0.924)	0.693 (0.557, 0.862)	*P* < 0.001
Model 2^c^	1	0.916 (0.766, 1.095)	0.753 (0.608, 0.931)	0.706 (0.566, 0.88)	*P* < 0.001
Model 3^d^	1	0.866 (0.724, 1.035)	0.737 (0.596, 0.911)	0.684 (0.549, 0.852)	*P* < 0.001
Model 4^e^	0.995 (0.993, 0.997)				*P* < 0.001

a Cox proportional hazards models were used to estimate the HRs (95% CIs) of all-cause mortality according to quartiles of serum selenium concentrations. Q, quartile.

b Model 1 was adjusted for age (continuous), sex (male or female), and race (non-Hispanic white or other).

c Model 2 was adjusted for age (continuous), sex (male or female), race (non-Hispanic white or other), family income–poverty ratio (>3.0, 1.1–3.0, and ≤1), BMI (≥30 or <30 kg/m^2^), serum triglycerides (≥200 or <200 mg/dL), serum total cholesterol (≥240 or <240 mg/dL), and serum uric acid (≥7 or <7 mg/dL).

d Model 3 was adjusted for age (continuous), sex (male or female), race (non-Hispanic white or other), family income–poverty ratio (>3.0, 1.1–3.0, and ≤1), BMI (≥30 or <30 kg/m^2^), serum triglycerides (≥200 or <200 mg/dL), serum total cholesterol (≥240 or <240 mg/dL), serum uric acid (≥7 or <7 mg/dL), diabetes (yes or no), hypertension (yes or no), and smoking status (smoker or never smoker).

e Continues model (each per 1% increase in serum selenium concentrations) adjusted by variables in model 3.

**Table 3:** CVD mortality according to quartiles of serum selenium concentrations among patients with CKD^a^.

**Characteristics**	**Serum selenium concentration (**μ**g/L)**				
	**Q1**<**156.1**	**Q2 156.1–181.7**	**Q3 181.7–201.5**	**Q4 201.5–734.8**	***P*** **-trend**
CVD mortality					
Number of deaths/total	185/776	74/768	40/762	37/767	336/3,063
Model 1^b^	1	0.878 (0.670, 1.153)	0.560 (0.394, 0.794)	0.535 (0.371, 0.771)	*P* < 0.001
Model 2^c^	1	0.865 (0.657, 1.139)	0.540 (0.380, 0.767)	0.525 (0.363, 0.758)	*P* < 0.001
Model 3^d^	1	0.834 (0.633, 1.100)	0.526 (0.370, 0.748)	0.513 (0.356, 0.739)	*P* < 0.001
Model 4^e^	0.992 (0.989, 0.996)				*P* < 0.001

a Competing risk models were used to estimate the HRs (95% CIs) of cardiovascular-cause mortality according to quartiles of serum selenium concentrations. Q, quartile.

b Model 1 was adjusted for age (continuous), sex (male or female), and race (non-Hispanic white or other).

c Model 2 was adjusted for age (continuous), sex (male or female), race (non-Hispanic white or other), family income–poverty ratio (>3.0, 1.1–3.0, and ≤1), BMI (≥30 or <30 kg/m^2^), serum triglycerides (≥200 or <200 mg/dL), serum total cholesterol (≥240 or <240 mg/dL), and serum uric acid (≥7 or <7 mg/dL).

d Model 2 was adjusted for age (continuous), sex (male or female), race (non-Hispanic white or other), family income–poverty ratio (>3.0, 1.1–3.0, and ≤1), BMI (≥30 or <30 kg/m^2^), serum triglycerides (≥200 or <200 mg/dL), serum total cholesterol (≥240 or <240 mg/dL), serum uric acid (≥7 or <7 mg/dL), diabetes (yes or no), hypertension (yes or no), and smoking status (smoker or never smoker).

e Continues model (each per 1% increase in serum selenium concentrations) adjusted by variables in model 3.

When subgroup analyses were based on age, sex, race, BMI, and smoking status, a similar correlation was found between selenium levels and all-cause mortality (Table 4). We also investigated the correlation between selenium levels and CVD mortality using subgroup analysis (Table 5). When subgroup analyses were based on age, sex, race, BMI, and smoking status, the correlation of selenium levels with all-cause mortality and CVD mortality was unchanged.

**Table 4:** Subgroup analyses of the associations between serum selenium concentrations and all-cause mortality among CKD patients^a^.

**Characteristics**	**Serum selenium concentration (**μ**g/L)**				
	**Q1**<**156.1**	**Q2 156.1–181.7**	**Q3 181.7–201.5**	**Q4 201.5–734.8**	***P*** **-trend**
Age, y					
<65	1	0.836 (0.523, 1.337)	0.605 (0.36, 1.015)	0.493 (0.296, 0.82)	0.003262
≥65	1	0.902 (0.742, 1.096)	0.798 (0.631, 1.008)	0.748 (0.585, 0.955)	0.007641
Sex					
Male	1	1.070 (0.846, 1.352)	0.718 (0.535, 0.964)	0.643 (0.478, 0.865)	0.00109
Female	1	0.675 (0.508, 0.897)	0.787 (0.576, 1.075)	0.797 (0.572, 1.111)	0.07929
Ethnicity (%)					
Non-Hispanic white	1	0.987 (0.786, 1.239)	0.739 (0.563, 0.97)	0.696 (0.529, 0.915)	0.00289
Other	1	0.701 (0.524, 0.937)	0.685 (0.485, 0.968)	0.644 (0.443, 0.936)	0.00813
BMI, kg/m^2^					
<30	1	0.922 (0.739, 1.151)	0.727 (0.547, 0.967)	0.765 (0.58, 1.009)	0.014546
≥30	1	0.794 (0.585, 1.077)	0.738 (0.531, 1.025)	0.560 (0.389, 0.807)	0.00147
Smoking status					
Never smoker	1	1.024 (0.777, 1.347)	0.816 (0.589, 1.13)	0.738 (0.522, 1.045)	0.06053
Smoker	1	0.798 (0.63, 1.01)	0.704 (0.531, 0.933)	0.651 (0.49, 0.866)	0.000728

a Cox proportional hazard models were used to estimate the HRs (95% CIs) of all-cause mortality according to quartiles of serum selenium concentrations. Results were adjusted for age (≥65 or <65), sex (male or female), race (non-Hispanic white or other), BMI (≥30 or <30 kg/m^2^), and smoking status (smoker or never smoker).

BMI, body mass index; Q, quartile.

**Table 5:** Subgroup analyses of the associations between serum selenium concentrations and CVD mortality among CKD patients^a^.

**Characteristics**	**Serum selenium concentration (**μ**g/L)**				
	**Q1**<**156.1**	**Q2 156.1-181.7**	**Q3 181.7-201.5**	**Q4 201.5-734.8**	***P*** **-trend**
Age, y					
<65	1	0.584 (0.283, 1.210)	0.466 (0.203, 1.070)	0.213 (0.080, 0.570)	<0.001
≥65	1	0.901 (0.669, 1.212)	0.599 (0.380, 0.822)	0.611 (0.415, 0.920)	<0.001
Sex					
Male	1	0.957 (0.664, 1.380)	0.491 (0.299, 0.806)	0.470 (0.284, 0.779)	<0.001
Female	1	0.667 (0.433, 1.028)	0.559 (0.336, 0.931)	0.588 (0.340, 1.018)	0.014
Ethic					
Non-Hispanic white	1	0.923 (0.652, 1.306)	0.489 (0.308, 0.777)	0.502 (0.317, 0.795)	<0.001
Other	1	0.694 (0.441, 1.092)	0.534 (0.305, 0.934)	0.482 (0.263, 0.884)	0.0064
BMI, kg/m^2^					
<30	1	0.949 (0.672, 1.342)	0.541 (0.331, 0.886)	0.565 (0.344, 0.929)	0.0039
≥30	1	0.664 (0.422, 1.044)	0.492 (0.298, 0.811)	0.425 (0.247, 0.730)	<0.001
Smoking status					
Never smoker	1	0.922 (0.608, 1.399)	0.826 (0.515, 1.324)	0.488 (0.268, 0.892)	0.022
Smoker	1	0.791 (0.546, 1.147)	0.355 (0.208, 0.605)	0.531 (0.335, 0.841)	<0.001

a Competing risk models were used to estimate the HRs (95% CIs) of all-cause mortality according to quartiles of serum selenium concentrations. Results were adjusted for age (≥65 or <65), sex (male or female), race (non-Hispanic white or other), BMI (≥30 or <30 kg/m^2^), and smoking status (smoker or never smoker).

BMI, body mass index; Q, quartile.

After excluding the special participants from the sensitivity analyses, the analyses were repeated using a fully adjusted model (model 3; Supplementary Table 1). The correlation of serum selenium concentration with all-cause mortality and CVD mortality was also unchanged after excluding participants who died in the 1st year during the follow-up period. Sensitivity analyses after excluding patients with CKD stages 1–2 or stages 3–5 indicated similar results.

## Discussion

To the best of our knowledge, this is the first prospective study to investigate the correlation between serum selenium concentration and mortality among patients with CKD. In this study, we revealed a correlation between serum selenium levels and all-cause mortality and CVD mortality after multivariable adjustment for age, sex, serum triglyceride concentration, serum total cholesterol concentration, serum uric acid concentration, BMI, smoking status, hypertension, and diabetes. Based on the results of various analyses, we confirmed the reliability of these findings.

Selenium is an essential nutrient and trace element necessary for human health that plays a crucial role in antioxidative metabolism and homeostasis (42). Selenium compounds from daily intake include selenite, selenocysteine, selenomethionine, and methylselenocysteine, which have different metabolic pathways and metabolites (43–47). Selenium can prevent the formation of atherosclerotic lesions and improve endothelial function by reducing superoxide generation and preventing mitochondrial DNA damage (48–52). Epidemiological evidence also confirmed the health benefits of high serum selenium levels in reducing deaths and CVD events. Jayedi et al.'s dose–response meta-analysis supported that serum selenium levels and daily intake of selenium were inversely correlated with all-cause mortality (53). Kuria et al. found that high selenium concentration was associated with a decreased risk of CVD incidence and mortality, and a higher dietary intake of selenium was associated with decreased cancer risk after adjusting for age, BMI, and smoking status (54, 55).

In the current study, we found that serum selenium levels were lower in older participants among patients with CKD. This was similar to the results of the study by Schiavon et al., who revealed that serum selenium levels decreased years before death (56). Our results indicate that the correlation of serum selenium levels with all-cause mortality and CVD mortality was not associated with age. Similar results were obtained when subgroup analyses were based on sex and race. Smoking is an important risk factor for various types of progressive diseases and may significantly increase oxidative stress (57, 58), The results of the current study revealed that higher concentrations of selenium also significantly correlated with the reduction of all-cause mortality and CVD mortality in these patients. Similar results were obtained when subgroup analyses were based on BMI. CKD stage is a key risk factor for CVD mortality in patients (59, 60). Our results revealed that in both CKD stages 1–2 and 3–5, higher levels of serum selenium were correlated with a reduction in all-cause mortality and CVD mortality.

In the general population, the serum selenium concentration associated with minimal mortality is 130–150 μg/L (61, 62). However, there is an intrinsic conflict between long-term health benefits based on epidemiological evidence and the controversial results of selenium supplementation studies in humans (14, 24–33, 62). Countering this mystery, there are some possible explanations for this problem. First, in laboratory studies, selenite is the most common form of selenium, whereas most human studies use SeMet as the most popular supplement (14). This difference in chemical form may explain the differences in health effects between the studies. Second, the metabolites of cells and *in vitro* studies are different from those of *in vivo* studies and human studies because of different environments and metabolic pathways. Third, excess selenium may harm normal glucose metabolic pathways and enhance the generation of peroxisome proliferator-activated receptor gamma (PPARγ) (63, 64). Therefore, previous studies have supported that those in the general population with serum selenium levels of 122 μg/L or higher may not need selenium supplementation (63). However, this is different for patients with CKD. Existing evidence indicates that serum selenium levels are lower in patients with CKD than those in the general population (28, 30, 31, 65). In the present study, we found that a higher concentration of serum selenium was correlated with decreased all-cause mortality and CVD mortality in patients with CKD. This is consistent with Ruiz et al.'s study, which found that adult patients on hemodialysis with lower selenium levels had a higher risk of death (65). In our study, the relationship between serum selenium levels and mortality among patients with CKD was different from the dual relationship confirmed in the general population. There are several potential explanations for this difference. First, the baseline levels of serum selenium and dietary intake of selenium in the two study populations were different (28, 30). Second, the oxidative stress levels in the two study populations were different, and abnormal oxidative stress levels were associated with abnormal selenium metabolism (1, 66). Third, serum selenium levels may not accurately reflect serum selenoprotein levels (14).

The present study has some strengths. First, the follow-up time of our study is long. Second, because the dietary intake of selenium in patients with CKD varied during the follow-up period, we used serum selenium levels as a better measure of selenium levels. Third, after adjusting for various covariates, the inverse correlation of selenium levels with all-cause mortality and CVD mortality was also evident, which confirmed the robustness of this association. Fourth, the subgroup analysis results indicated that the association existed generally in these subgroups of patients with CKD, which may reveal the universality of this association.

The present study has some limitations. First, although the current study was a cohort study, every participant's follow-up data were limited. Second, there was only one serum selenium concentration data point at the baseline, but the dietary intake of selenium changed gradually during the follow-up period, especially when the stages of CKD in patients progressed over years; thus, the baseline level of serum selenium may not accurately reflect the long-term level of serum selenium. Third, the dietary intake of selenium varies worldwide, and the level of serum selenium in our study participants was relatively high (14, 62). Thus, our results may not accurately reflect the association between selenium status and mortality in populations with lower serum selenium levels. Fourth, selenium's biological activities are mediated by selenium metabolites such as selenoproteins. Accurate measurement of selenoprotein and other selenium metabolites will reveal the underlying metabolism between serum selenium and mortality. Finally, the correlation is not causation; therefore, our results cannot prove causality because of the inherent limitations of observational studies.

## Conclusion

Higher serum selenium concentration was independently associated with a decreased risk of all-cause mortality and CVD mortality in patients with CKD.

## Data availability statement

The original contributions presented in the study are included in the article/Supplementary material, further inquiries can be directed to the corresponding authors.

## Ethics statement

This study involved secondary data analysis of a nationally representative publicly available dataset. The study we conducted was exempted from institutional review for this reason.

## Author contributions

DZ and QZ analyzed the data and drafted the manuscript. TL and TS designed the study and revised the manuscript. All authors approved the final version of the manuscript, ensured the accuracy, integrity of the study, and agree to be accountable for all aspects of the study.
