# Supplementary material for: Higher serum selenium concentration is associated with lower risk of all-cause and cardiovascular mortality among individuals with chronic kidney disease: A population-based cohort study of NHANES
Source: Front Nutr. 2023 Mar 31;10:1127188. doi: 10.3389/fnut.2023.1127188 (PMC10102510; doi:10.3389/fnut.2023.1127188)
Supplement: Supplementary file 1 [file Table_1.docx]

**Supplementary TABLE 1 Results of sensitivity analyses of the associations between serum selenium concentrations and mortality^1^**

| Quartiles of serum selenium level | All-cause mortality | | | CVD mortality | | |
| --- | --- | --- | --- | --- | --- | --- |
|  | Event/Total | HR (95%CI) | P value | Event/Total | HR (95%CI) | P value |
| Excluding participants died in first follow-up 12 months | | | | | | |
| Q1 | 444/732 | 1 |  | 169/732 | 1 |  |
| Q2 | 152/743 | 0.866(0.716, 1.047) | 0.1370 | 64/743 | 0.874(0.651, 1.172) | 0.37 |
| Q3 | 105/748 | 0.778(0.621, 0.974) | 0.0289 | 36/748 | 0.576(0.398, 0.834) | 0.0035 |
| Q4 | 101/758 | 0.748(0.595, 0.942) | 0.0136 | 34/758 | 0.562(0.383, 0.825) | 0.0032 |
| CKD stage 1–2 participants | | | | | | |
| Q1 | 147/304 | 1 |  | 64/304 | 1 |  |
| Q2 | 59/374 | 0.894(0.718, 1.113) | 0.3174 | 20/374 | 0.972(0.701, 1.348) | 0.86 |
| Q3 | 44/410 | 0.700(0.537, 0.912) | 0.0082 | 16/410 | 0.499(0.320, 0.779) | 0.0022 |
| Q4 | 50/455 | 0.618(0.463, 0.825) | 0.0011 | 15/455 | 0.542(0.342, 0.859) | 0.0092 |
| CKD stage 3–5 patients | | | | | | |
| Q1 | 331/462 | 1 |  | 121/462 | 1 |  |
| Q2 | 118/394 | 0.838(0.614, 1.144) | 0.2659 | 54/394 | 0.601(0.360, 1.004) | 0.052 |
| Q3 | 75/352 | 0.826(0.574, 1.186) | 0.3001 | 24/352 | 0.539(0.304, 0.957) | 0.035 |
| Q4 | 60/312 | 0.850(0.598, 1.208) | 0.3652 | 22/312 | 0.483(0.264, 0.882) | 0.018 |

^1^ Data are presented as HR (95% CI). Adjusted for age (continuous), sex (male or female), race (non-Hispanic white or other), family income–poverty ratio (>3.0, 1.1–3.0, ≤1), BMI (≥30, or <30kg/m2), serum triglycerides (≥200mg/dL, or <200mg/dL), serum total cholesterol (≥240mg/dL, or <240mg/dL), serum uric acid (≥7mg/dL, or <7mg/dL), diabetes (yes or no), hypertension (yes or no), smoking status (smoker or never smoker)
